# A Narrative Review of Chondrocalcinosis: Clinical Presentation, Diagnosis, and Therapies

**DOI:** 10.7759/cureus.60434

**Published:** 2024-05-16

**Authors:** Soo Yeon Kim, Sana Afroz, Heather Gillespie, Christina Downey

**Affiliations:** 1 Rheumatology, Loma Linda University Health, Loma Linda, USA

**Keywords:** inflammatory arthritis, chondrocalcinosis, acute cpp arthritis, crystal arthritis, calcium pyrophosphate deposition disease (cppd)

## Abstract

Calcium pyrophosphate deposition disease is categorized into radiographic chondrocalcinosis, acute calcium pyrophosphate arthritis, chronic calcium pyrophosphate arthritis, and osteoarthritis with calcium pyrophosphate deposition. These entities collectively are characterized by the deposition of calcium into joints, which then may cause localized and systemic inflammation, resulting in pain and swelling in the affected joints. Patients with the *ANKH* gene are more susceptible to the development of CPP arthritis as are those with primary hyperparathyroidism, hypomagnesemia, and hemochromatosis. Radiographic chondrocalcinosis is asymptomatic. Acute calcium pyrophosphate arthritis results in self-limited periods of joint pain and swelling in the affected joint. Along with localized inflammation, there may also be systemic inflammation characterized by fever and elevated inflammatory markers. Chronic calcium pyrophosphate arthritis results in periods of quiescence interrupted by flares that are identical to acute periods of disease. Osteoarthritis associated calcium pyrophosphate arthritis presents with chronic pain well described in osteoarthritis with periods of acute flares. In 2023, a joint effort by the American College of Rheumatology and the European League Against Rheumatism developed guidelines meant to aid in the recognition of calcium pyrophosphate deposition diseases. The diagnosis is made if there is proof of either crowned dens syndrome or synovial fluid analysis demonstrating calcium pyrophosphate crystals or when more than 56 points are summed utilizing the criteria described in the guidelines. Radiographic chondrocalcinosis requires no therapy. Acute calcium pyrophosphate arthritis is treated with the goal of aborting the flare. Treatment options include nonsteroidal anti-inflammatory drugs (NSAIDs), colchicine, oral corticosteroids, parenteral corticosteroids, intraarticular corticosteroids, IL-1 inhibitors, or parenteral adrenocorticotropic hormone (ACTH). The goal in treatment for chronic calcium pyrophosphate arthritis is the suppression of acute flares. The drugs used for acute flare treatment may be given as maintenance therapy with the additional options of methotrexate and hydroxychloroquine.

## Introduction and background

In 1952, Kohn, Hughes, McMarty, and Faures from Pennsylvania reported in their publication, “In the course of examining over 200 consecutive specimens of synovial fluid with phase contrast and polarized light microscopy, a collection of fluids from seven patients were obtained containing significant amounts of an unknown crystalline material” [1]. Initially suspecting gout, they soon observed distinctions in these arthritic manifestations that set them apart from the typical cases of gout, which is caused by monosodium urate crystals. They documented, “...the serum urate levels were usually normal... and most important, the nature of the crystalline material observed in the groups were different chemically” [1]. Their discovery led to the identification of calcium pyrophosphate deposition (CPPD) disease.

CPPD is the term used for several entities that include both acute and chronic calcium pyrophosphate (CPP) crystal arthritis, radiographic deposition of CPP crystals (radiographic chondrocalcinosis), and osteoarthritis with CPPD [2]. Chondrocalcinosis refers to cartilaginous calcifications most frequently seen on radiographs of the shoulders, wrists, and knees but may affect nearly any joint of the body and may be visualized on many imaging modalities. This form of disease may be asymptomatic. Acute CPP arthritis was previously known as pseudogout, but the term is no longer used. Development of CPPD is positively correlated with advancing age, previous joint injuries, osteoarthritis, primary hyperparathyroidism, hypomagnesemia, and hemochromatosis [3]. When CPP arthritis is present in a relatively younger person, workup for these entities should be considered. These entities collectively are characterized by the deposition of calcium into joints, which then may cause localized and systemic inflammation, resulting in pain and swelling in the affected joints. Symptoms include localized and systemic inflammation characterized by fever and elevated inflammatory markers. Chronic CPP arthritis results in periods of quiescence interrupted by flares that are identical to acute periods of disease. Osteoarthritis-associated CPP arthritis presents with chronic pain well described in osteoarthritis with periods of acute flares [2,3].

To aid in the uniformity of patient pools in future research, classification guidelines were developed to aid in identifying patients with CPP arthritis and CPPD disease. There are definitions of radiographic identification of CPPD on imaging modalities, such as radiographs, computed tomography (CT), dual-energy CT (DECT), and ultrasound (US), published in 2023. Both the classification criteria and consensus statement on imaging definitions of CPPD were developed by international multidisciplinary working groups. The diagnosis of CPPD is to be made if there is proof of either crowned dens syndrome or synovial fluid analysis demonstrating calcium pyrophosphate crystals or when more than 56 points are summed utilizing the criteria described in the guidelines.

The exact prevalence of CPPD is not well-established, although it is estimated to affect >10% of older adults [4]. In the United States, it is estimated that CPPD arthritis affects around 4-7% of people over the age of 60. A national cross-sectional study conducted among 25,157 U.S. veterans of age 68 ± 12.3 years, composed of 95% male subjects, reported an estimated prevalence of 0.52% [5]. Some studies report that the radiographic evidence of chondrocalcinosis can be observed in as many as 4% of individuals aged 40 or older with the prevalence tending to be higher in older individuals [2,3,5].

In addition to the risk factors listed above, there are genetic variants that predispose one to the development of CPPD. One such mutation is the *ANKH *gene, which is responsible for regulating inorganic pyrophosphate transport [6]. In 2023, a joint effort by the American College of Rheumatology (ACR) and the European League Against Rheumatism (EULAR) produced new guidelines to classify CPPD as a first step toward further research into this disease. In 2011, the EULAR guidelines made nine recommendations for CPPD management, but this guidance was published prior to the widespread use of biological therapies for CPPD management [7].

Pathophysiology

The process of CPP crystal formation in the articular joint has not been completely elucidated. Nevertheless, it appears that the CPP crystals in the cartilage trigger inflammatory pathways and lead to direct damage to the articular cartilage. The CPP crystals deposit in the pericellular matrix of cartilage called pericellular matrix vesicles derived from chondrocytes and osteoblasts. These vesicles concentrate calcium and phosphate in the lumen resulting in crystal generation [3,8,9]. The crystal deposition is regulated by the equilibrium of phosphate (Pi) to inorganic pyrophosphate (PPi). The production of extracellular PPi by chondrocytes is a normal, non-pathological process, and an appropriate physiological concentration of PPi is known to prevent the formation of basic calcium phosphate (BCP), which can lead to osteoarthritis [8]. However, excessive PPi concentration promotes the formation of CPP crystals in the cartilage. Several mechanisms of Pi and PPi homeostasis have been proposed.

Nucleotide pyrophosphatase phosphodiesterase (NPP1) is an enzyme that is involved in bone mineralization, insulin receptor signaling, malignancy, and immune processes [10]. It hydrolyzes adenosine triphosphate (ATP) to adenosine monophosphate (AMP) and diphosphate, yielding PPi from extracellular ATP. Although the regulation mechanism of this enzyme has been understood, deficiency of NPP1 has been associated with arterial calcification of infancy in humans and calcium phosphate deposition in the joints and spine of murine models [11]. NPP1 has been explored as a potential therapeutic target for CPPD, diabetes, and cancer. However, the application of NPP1 inhibition for CPPD treatment is under further investigation due to its association with possible undesirable side effects, such as hypo-mineralization of the long bones and calcification of soft tissues [8,10]. 

Tissue-nonspecific alkaline phosphatase (TNAP) is another enzyme well known for mineralization of the bone. It is an antagonist to NPP1 that hydrolyzes ATP and PPi to Pi. The mechanism of how the two enzymes, TNAP and NPP1, collaborate in bone mineralization is uncertain. However, studies have demonstrated that a reduced TNAP activity, as observed in congenital hypophosphatasia, can be associated with CPP crystal deposition in the articular cartilage [11,12].

Discovered in 2000, the *ANKH* gene encodes a multi-pass transmembrane protein called ankyrin, a protein responsible for transporting PPi across the membrane, from the intracellular to the extracellular compartment in various cell types [8,11,13,14]. Upregulation of the *ANKH* gene has been associated with CPPD and a potential therapeutic target. Probenecid, which was FDA-approved in 1979 for the treatment of gout, was also shown to inhibit *ANKH*-mediated PPi transport [13,15].

The nucleotide-binding and oligomerization (NOD) domain-like receptor (NLR) family pyrin domain (PYD)-containing (NLRP), also known as NACHT, LRR, and PYD domain-containing protein (NALP3), was discovered in the early 2000s and has gained attention due to its association with a wide spectrum of disorders, such as CPPD, gout, systemic juvenile idiopathic arthritis, pulmonary fibrosis, familial Mediterranean fever, Muckle-Wells syndrome, Alzheimer’s syndrome, type 2 diabetes, and pyoderma gangrenosum [16]. Dysregulation of NLRP3 activity has been found to increase IL-1β activity. Roughly speaking, NLRP sense pathogen-associated molecular patterns (PAMPs) or damage-associated molecular patterns (DAMPs) lead to the generation of inflammasomes, which subsequently activate caspase-1. Caspase-1 increases IL-1β secretion. Therefore, an inhibition of any part of this cellular pathway has been proposed and explored as a novel therapeutic target for CPP arthritis. IL-1 inhibitors, such as anakinra, rilonacept, and canakinumab, have been developed. Inflammasome inhibitors, such as dapansutrile, and caspase-1 inhibitions are being explored [16].

Clinical presentation

Clinically, CPPD may present as asymptomatic radiographic calcium pyrophosphate deposition incidentally found on radiographs obtained for other purposes. This presentation often co-exists in older patients with comorbid osteoarthritis and affects the knees most commonly, but may also be seen in the wrist, shoulder, symphysis pubis, and other joints [17]. Ultrasonography is a helpful tool to identify calcium deposition, most reliably found in the triangular fibrocartilage of the wrist, and acromioclavicular joint although crystal deposition may also be seen in the metacarpophalageal joints and hip joints [18].

**Figure 1:**
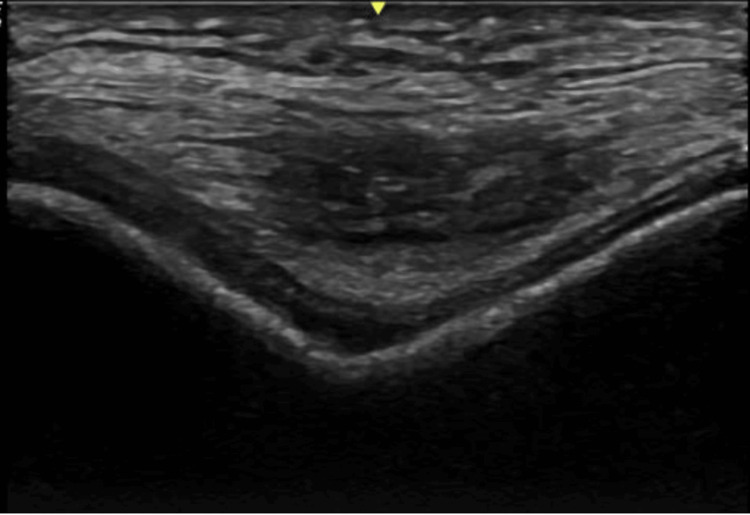
Chondrocalcinosis seen within the knee joint on ultrasound with knee maximally flexed and transducer applied transversely.` Image courtesy of Dr. Woo Young Kim (rheumatologist).

Figure 1 shows a chondrocalcinosis seen within the knee joint with the knee maximally flexed and the transducer applied transversely.

Acute presentations of CPP arthritis are clinically similar to acute gout flares, manifested by severe pain, redness, warmth, swelling, and tenderness. Flares are most likely to be monoarticular, but oligoarticular flares do occur. Accompanying signs and symptoms may include fever, elevated inflammatory markers, leukocytosis, thrombocytosis, and elevated ferritin. When compared to acute gout, the course of acute CPP arthritis may be drawn out, taking longer to peak and longer to subside. Between attacks, there is a period of quiescence that is symptom-free [19]. Performing a joint aspiration is often required to rule out a septic joint as the clinical presentation may be identical and can occur simultaneously in the same joint [20].

As the diagnosis has become more sophisticated and has started to encompass specialized imaging modalities, such as DECT, our understanding of the clinical spectrum has expanded, and acute CPP arthritis has been described in the cervical dens, known as crowned dens syndrome [21]. Crowned dens syndrome presents as severe neck pain and stiffness with elevated inflammatory markers. Care must be taken to rule out infections, such as meningitis, even when the characteristic findings are seen on DECT [22]. Findings seen on DECT of the cervical spine include linear calcific deposits less dense than bone and seen as two parallel lines in the atlas' transverse ligament [21].

Chronic CPP arthritis is characterized by chronic, symmetric, deforming polyarthritis and may be confused with rheumatoid arthritis as it commonly affects the metacarpal-phalangeal joints and wrists; however, chronic CPP arthritis does not produce the erosions seen in rheumatoid arthritis on imaging. Patients with chronic CPP arthritis may also have flares of acute CPP arthritis. In some patients, chronic CPP arthritis may resemble polymyalgia rheumatica or Charcot joint, both of which may be challenging to differentiate from their mimickers [19]. Patients presenting with primarily bilateral destruction of the subtalar, talonavicular, tarsometatarsal, and/or naviculocuneform joints in the absence of diabetes or neuropathy should undergo DECT or other advanced imaging to detect calcium pyrophosphate deposition [23]. DECT is particularly useful because it can differentiate monosodium urate crystals from CPPD crystals [24]. With experience and training, a musculoskeletal ultrasonographer may also be able to differentiate monosodium urate crystals from CPPD crystals in some joints [25].

A rare entity described in case reports or case series is tumoral CPPD, which may be confused with the calcinosis of dermatomyositis or scleroderma, or malignancy-associated calcinosis. Most commonly, tumoral CPPD deposition occurs in the temporomandibular joints, but there are cases of peripheral and spinal tumoral CPPD [26]. 

Diagnosis

CPPD arthritis should be suspected in patients who develop joint pain, swelling, or tenderness that cannot be explained by an alternative diagnosis, such as osteoarthritis, rheumatoid arthritis, or gout. If crowned dens syndrome is diagnosed or if synovial fluid aspiration demonstrates CPP crystals in a symptomatic joint, these are adequate to diagnose the condition. Crowned dens syndrome is defined as presence of both clinical and imaging features, both of which must be met for diagnosis. Clinical features include an acute or subacute onset of severe pain localized to the upper neck with elevated inflammatory markers, limited rotation, and often fever. Mimicking conditions, such as polymyalgia rheumatica and meningitis, should be excluded. Imaging features include conventional CT showing calcific deposits, typically linear and less dense than cortical bone, in the transverse retro-odontoid ligament (transverse ligament of the atlas), often with an appearance of two parallel lines in axial views. Calcifications at the atlantoaxial joint, alar ligament, and/or pannus adjacent to the tip of the dens are also characteristic. DECT features of CPP deposition include a dual-energy index between 0.016 and 0.036 [4].

In 2023, a joint effort by the ACR and EULAR produced clinical classification criteria with the aim of providing a framework for research study participant identification. However, in practice, these classification criteria are used to guide clinical diagnosis as well. The goal of research studies is to provide a pure patient population, but in clinical practice, the goal is to ensure that all patients with the disease are captured, which is the fundamental difference between classification and diagnosis criteria. As there are no diagnostic guidelines for many rheumatic diseases, classification criteria are used a guide. If the previously mentioned criteria are not met immediately, then a score of greater than 56 on the ACR/EULAR classification criteria is indicative of CPPD. The criteria do not need to be present at the same time to count and exclusion criteria should be evaluated carefully prior to applying the classification criteria. Domains included are age, time course of disease, site affected, and imaging and synovial fluid analysis findings. Application of these classification criteria has a sensitivity of 96.5% and a specificity of 92.5% in the derivation cohort (190 CPPD cases, 148 mimickers), whereas sensitivity was 99.2% and specificity was 92.5% in the validation cohort (251 CPPD cases, 162 mimickers) [4].

Specific imaging modalities beyond plain film radiography have been studied for their usefulness in the recognition of CPP deposition, particularly US and DECT. A review of 37 studies reported US detected CPPD with a sensitivity ranging from 0.34 (CI 0.16-0.58) and 0.77 (0.63-0.87). The specificity varies between 0.92 (0.16-1.00) and 1.00 (0.89-1.00). When adjusting for synovial fluid analysis as a reference standard, the pooled sensitivity of US was 0.87 (95% CI 0.76-0.99) and specificity 0.98 (0.96-1.00). When the reference standard was radiography, the sensitivity of US was 0.58 (95% CI 0.09-1.00) and specificity was 0.84 (95% CI 0.52-1.00) [18].

In 2023, an international multidisciplinary working group developed a framework for diagnosing CPDD based on imaging modalities, including US. Crystal deposition should be in the fibrocartilage or hyaline cartilage to qualify as CPP deposition. This deposition is described as hyperechoic deposits with variable shape and size and do not create posterior shadowing. US has the advantage of assessing the response to motion, and CPP deposition in the cartilaginous structures should move as the joint is moved. Hyperechoic deposits may also be visualized on US in the synovial membrane, joint capsule, or tendons, and these deposits do not move when the joint is moved. The main drawbacks of ultrasonography use for CPP arthritis diagnosis is that the accuracy relies on the experience of the operator and the inability distinguish CPP deposition from basic calcium deposition disease [21]. 

DECT utilizes two different X-ray energy levels to characterize soft tissues and joints. It has become increasingly utilized in the evaluation of crystal arthropathy due to its ability to characterize the crystals as monosodium urate or CPPD. DECT showed a comparable sensitivity and specificity of ultrasonography in the diagnosis of acute gout and acute CPP crystal arthritis in a retrospective study. Surprisingly, as opposed to DECT, radiographs had the lowest sensitivity and specificity [27]. To explore additional utilities of DECT, a single-center cross-sectional study was conducted to determine the utility of DECT in the diagnosis of early CPPD of the knees. The participants were composed of crystal-proven CPPD (n = 50) or a control (n = 82). The study concluded that DECT is not suitable for the early detection of CPP when other forms of crystal deposition are present that may be visible on the conventional CT but that DECT could be most appropriately utilized for the characterization of the crystal compositions [28]. The utility of DECT in characterizing crystals was further supported in recent studies [28]. A prospective study of 13 patients aged 57 ± 18 years with symptoms of calcific periarthritis or tendinitis showed DECT’s capacity to distinguish BCP and calcium pyrophosphate in and around joints in vivo. The study compared findings on DECT of the patients with BCP crystal arthritis, whose diagnosis was confirmed via surgical or percutaneous needle aspiration, with those of patients with CPPD whose diagnosis was confirmed via synovial fluid analysis. Distinguishing BCP crystal arthritis and CPPD using conventional methods, such as radiography or CT, has been challenging due to similar attenuation, and the type of calcium crystal has been speculated based on the anatomical location. However, the study showed DECT’s ability to distinguish BCP and CPP using DECT and showed a promising future role of DECT in challenging clinical cases or optimization of the treatment course when the identity of the crystal is not readily available by other means [28].

## Review

Treatment

Guidance for the treatment of CPP arthritis was published by the EULAR in 2011, prior to the use of biological therapy in the management of both acute and chronic forms of CPP arthritis [29]. Treatment of CPP arthritis differs depending on the presentation of the disease and the comorbid conditions present in the patient. Asymptomatic radiographic CPPD does not require any therapy.

Acute CPPD arthritis should be treated immediately to reduce ongoing inflammation and damage to the involved joint and soft tissue structures. Pharmacologic treatments are often used in tandem with other modalities, such as ice packs and joint rest. Pharmacologic management of acute CPP arthritis includes non-steroidal anti-inflammatory drugs (NSAIDs), such as indomethacin and ibuprofen [30]. These are chosen for their short onset of action but should be used for the shortest time needed to suppress acute inflammation due to their well-known potential adverse effects, particularly in the age group most likely to be afflicted by CPP arthritis. An alternative to NSAIDs is oral colchicine, which should be taken as quickly within 24 hours of the onset of symptoms [31].

Colchicine, found in the seeds and flowers in a variety of *Colchicum autumnale*, is an alkaloid compound with a narrow therapeutic index, requiring monitoring of symptoms of colchicine toxicity, such as nausea, vomiting, diarrhea, and abdominal pain, especially in the setting of renal or hepatic impairment [32]. Its mechanism of action involves blocking microtubule polymerization, interfering with the metaphase of the cells, and inhibiting neutrophil chemotaxis and innate immune responses [33,34]. A small prospective cohort study composed of 12 patients, conducted in 1978 in Spain, reported that the frequency of acute CPP crystal attacks was reduced to an average of 2.4 from 9.3 attacks (P < 0.05) after treatment with daily 1 mg of oral colchicine for one year [33,35]. Other studies have also shown the utility of colchicine as a prophylactic agent against recurrent acute CPP arthritis at a dose of 0.6 mg twice daily [36].

In cases where NSAIDs and colchicine cannot be used, corticosteroids may be considered for acute flares. Corticosteroids work quickly to reduce inflammation and pain and may be given orally, intramuscularly, intravenously, or intraarticularly. In a study conducted among 28 hospitalized patients, joint aspiration combined with intra-articular injection of corticosteroid provided faster relief, in approximately 24-48 hours, as opposed to joint aspiration with oral NSAIDs, which took two to seven days for the resolution of synovitis [37]. When a single joint is affected, intraarticular steroids offer relief with few systemic side effects, but in oligoarticular presentations, systemic therapy is required. Due to the adverse effects seen with chronic steroid use, a short course is preferred to abort attacks. Even in short-term use with systemic corticosteroids, patients should be advised that they may experience interference with sleep, psychological changes, hyperglycemia, hypertension, glaucoma, or appetite changes.

The EULAR treatment guidelines also cite parenteral ACTH as an option for patients with acute CPP arthritis, but the adverse effect profile was similar to that of corticosteroids, hypokalemia, hyperglycemia, fluid retention, and rebound arthritis. To the authors’ knowledge, a cost-effectiveness analysis has not been completed examining the use of parenteral ACTH in the treatment of CPPD disease when compared to parenteral corticosteroids, but this has been studied in other diseases. In a study of pediatric patients with West syndrome, prednisolone was found to be half the cost of parenteral ACTH, and ACTH only performed slightly better in treatment response [38].

When colchicine, NSAIDs, and corticosteroids are not viable options for abortive therapy, IL-1 inhibitors may be used. Anakinra is given as a daily subcutaneous injection, which works rapidly to decrease inflammation and pain. In a retrospective review of anakinra use in hospitalized patients with a crystal or imaging-proven diagnosis of CPP acute arthritis, the adverse effect profile was minimal and not substantially different from those described in the literature utilizing other modalities of treatment for acute attacks. Attacks were mitigated within four days of the use of anakinra [39].

A double-blinded randomized controlled trial showed that anakinra was more efficacious than oral steroids in treating acute CPP arthritis. This study compared a three-day course of anakinra and prednisone. Eight participants received 100 mg anakinra, and seven participants received oral prednisone 30 mg for three days. The outcome was measured using the visual analog scale (VAS). The study noted a statistically significant decrease in VAS score in the anakinra group compared to the steroid group at 72 hours. The study was not completed due to poor recruitment but showed a faster improvement of pain with Anakinra compared to prednisone [40]. 

Similarly, a retrospective study conducted on hospitalized patients with acute CPP arthritis showed the utility of anakinra in reducing the pain VAS in 79% of patients (n = 14) after two to four doses. One patient reported a rash that resolved with diphenhydramine [41]. Another study conducted by Molto and Olive of five retrospective chart reviews showed that crystal-proven CPP arthritis responded well to a three-day treatment with anakinra after failure or contraindication to NSAIDs, corticosteroids, and colchicine [42].

Other IL-1 inhibitors, such as rilonacept, which is longer acting, and canakinumab, a fully humanized anti-IL-1beta monoclonal antibody, have been studied for the management of refractory gout. Currently, there are no randomized controlled trials showing safety or efficacy in CPP arthritis [43,44]. OLT1177, a novel NLRP3 inflammasome inhibitor, has been shown to reduce synovial IL-1β levels and joint swelling in murine models and has gained attention as a treatment target for crystal arthritis [29]. In a recent open-label, phase 2a trial, oral dapansutrile of varying doses from 300 to 2000 mg daily demonstrated more than 50% reduction in joint pain on day 3 regardless of the dosage. The most common adverse events were ironically the development of gout or gout flares in other joints [45]. 

Disease-modifying anti-rheumatic drugs (DMARDs), used for decades in the treatment of other types of inflammatory arthritides, have been studied to varying degrees with mixed results in chronic CPP arthritis. Methotrexate (MTX) is a structural analog of folate that inhibits DNA synthesis and hence cellular replication and is one of the oldest DMARDs, in use since the 1950s [46]. Its use in the management of CPP arthritis remains unclear as studies have shown contrasting views [33,47,48]. In an observational study conducted involving 10 patients of median age 67.3 years with synovial fluid-proven CPP arthritis refractory to NSAIDS, colchicine, or glucocorticoids, MTX showed a positive response. On a scale of 0 to 10, 0 equivalent to no effect and 10 being complete resolution of symptoms, patients achieved a median score of 7.4, on a varying dose of MTX (7.5-25 mg weekly; median 13.75 mg weekly) during a varying duration of the treatment period (six to 48 months; median 15.2 months) [47].

However, a double-blinded crossover randomized control trial conducted in Switzerland perceived no strong effect of MTX in chronic or recurrent CPP arthritis. The patients received either weekly subcutaneous injections of 15 mg MTX or placebo for three months, followed by a wash-out period of two months, and a crossover treatment for another three months. The outcome was measured by DAS44, which is a disease activity scoring system composed of a number of swollen joints, a number of tender joints, and the erythrocyte sedimentation rate. The study found no significant difference between the DAS44 of treatment and placebo groups at three months (p = 0.43) [49]. Similarly, hydroxychloroquine (HCQ) has shown mixed results in the management of CPP arthritis [50,51].

While not first line, HCQ has been used in CPP arthritis although it has not been extensively studied. In one double-blind randomized crossover trial, patients received a dose between 100 mg and 400 mg daily. Results showed that 76% of patients in the HCQ group achieved a 30 percent reduction in the number of flares compared to 32% in the placebo group [50]. HCQ is originally an anti-malarial drug, and its usefulness in CPPD is linked to its capability to immunomodulate and reduce inflammation. HCQ blocks the activity of T cells and reduces the release of cytokines, such as IL-1, IL-6, and TNF. One potential theory is that the CPP flares are reduced by potentially stabilizing phagolysosomes to suppress NLRP3 inflammasome activation triggered by crystal uptake [52]. Both MTX and HCQ were recommended as treatment options in the 2011 EULAR guidelines [29]. 

Treatment is an area of ongoing research and novel agents are currently in various stages of development. Inhibition of IL-1 appears to be the most promising mechanism of action for the development of anti-CPPD agents in the future. Acute CPP arthritis is treated with the goal of aborting the flare. Treatment options include NSAIDs, colchicine, oral corticosteroids, parenteral corticosteroids, intraarticular corticosteroids, IL-1 inhibitors, or parenteral ACTH. The goal in treatment for chronic CPP arthritis is the suppression of acute flares. The drugs used for acute flare treatment may be given as maintenance therapy with the additional options of MTX and HCQ. The choice of agent is driven by patient comorbidities, the anticipated length of therapy, the cost and availability of the chosen agent, and the patient’s ability to tolerate the chosen therapy.

## Conclusions

CPP arthritis can be acute or chronic and can cause significant disability and pain. There are no known ways to prevent or cure CPP arthritis, although flares can be treated acutely and suppressed with certain medications. Future directions include genetic investigations to determine if patients with the ANKH gene can modify the likelihood of developing CPP arthritis, either through pharmacologic, genetic, or lifestyle interventions. More research is needed to assess whether management of primary hyperparathyroidism, hypomagnesemia, and hemochromatosis results in a change in the likelihood of developing CPP arthritis. More work needs to be done to determine if CPP arthritis increases the risk of cardiovascular disease as is the case with other inflammatory arthritides.
